# NDAMDA: Network distance analysis for MiRNA‐disease association prediction

**DOI:** 10.1111/jcmm.13583

**Published:** 2018-03-13

**Authors:** Xing Chen, Le‐Yi Wang, Li Huang

**Affiliations:** ^1^ School of Information and Control Engineering China University of Mining and Technology Xuzhou China; ^2^ School of Mathematics and Statistics Wuhan University Luojiashan Wuchang China; ^3^ Business Analytics Centre National University of Singapore Singapore Singapore

**Keywords:** adjusted network distance, association prediction, disease, microRNA, network integration

## Abstract

In recent years, microRNAs (miRNAs) are attracting an increasing amount of researchers’ attention, as accumulating studies show that miRNAs play important roles in various basic biological processes and that dysregulation of miRNAs is connected with diverse human diseases, particularly cancers. However, the experimental methods to identify associations between miRNAs and diseases remain costly and laborious. In this study, we developed a computational method named Network Distance Analysis for MiRNA‐Disease Association prediction (NDAMDA) which could effectively predict potential miRNA‐disease associations. The highlight of this method was the use of not only the direct network distance between 2 miRNAs (diseases) but also their respective mean network distances to all other miRNAs (diseases) in the network. The model's reliable performance was certified by the AUC of 0.8920 in global leave‐one‐out cross‐validation (LOOCV), 0.8062 in local LOOCV and the average AUCs of 0.8935 ± 0.0009 in fivefold cross‐validation. Moreover, we applied NDAMDA to 3 different case studies to predict potential miRNAs related to breast neoplasms, lymphoma, oesophageal neoplasms, prostate neoplasms and hepatocellular carcinoma. Results showed that 86%, 72%, 86%, 86% and 84% of the top 50 predicted miRNAs were supported by experimental association evidence. Therefore, NDAMDA is a reliable method for predicting disease‐related miRNAs.

## 1 INTRODUCTION

MicroRNAs (miRNAs) are a class of non‐coding RNAs which play regulatory roles in gene expressions by binding to the complementary regions of messenger transcripts to repress their translation or regulate degradation.^1^, ^2^, ^3^ Asince the lin‐4 and let‐7 were discovered in *Caenorhabditis elegans*,^4^ over 30 000 mature miRNAs have been found from 206 species.^5^ Furthermore, accumulating evidence shows that miRNA constitutes one of the most important components of cells and is involved in nearly all biological processes, including cell growth,^6^ immune reaction,^7^ cell proliferation and differentiation,^8^, ^9^ cell development,^6^ cell cycle regulation,^10^ inflammation,^11^ apoptosis 12 and stress response.^6^, ^13^ It is also reported that the abnormality of miRNAs is connected with various human diseases, particularly cancers.^14^, ^15^ Identifying disease‐related miRNAs is an important biomedical research field, which benefits the understanding of disease pathogenesis at the molecular level and the design of molecular tools for disease diagnosis, treatment and prevention.

In parallel with much efforts being made to identify novel miRNAs, the research community is also interested in predicting and validating miRNAs’ associations with diseases. Using experimental methods to uncover such associations is typically costly and time‐consuming. Fortunately, taking the advantage of vast biological data for miRNAs, computational methods can be an efficient complement to experimental studies. By far, existing computational methods can be broadly divided into 2 categories: (i) those constructing networks and applying the corresponding network‐based algorithms and (ii) those utilizing machine learning.

Inspired by the idea that functionally similar miRNAs tend to be related with phenotypically similar diseases, Jiang et al^16^ constructed a scoring system based on a discrete hypergeometric probability distribution. This method was not particularly satisfying because 2 miRNAs may be functionally related when their target genes, instead of overlapping significantly, reside in the same cellular pathways or functional modules. Some researchers developed their methods by involving other functional elements. For example, Mork et al^17^ combined miRNA‐protein and protein‐disease associations and proposed a novel model named miRNA‐protein‐disease (miRPD). This method took proteins into consideration, but finding enough miRNAs and diseases associated with the same proteins for the analysis was a difficult task. Shi et al^18^ presented a method to identify miRNA‐disease associations based on the assumption that a disease tends to be associated with miRNAs whose target genes also have associations with this disease. They carried out a random walk analysis on a protein‐protein interaction (PPI) network, and the analysis took into account the global network distance measure and the functional links between miRNAs’ targets and disease genes. However, the methods mentioned above had two common drawbacks: the high false‐positives and false‐negatives in miRNA‐target interactions and the incompletion of the disease‐gene association network.

To overcome such drawbacks, researchers developed computational models without relying on miRNA‐target interactions. Chen et al^19^ put forward RWRMDA, the first global network similarity‐based model, to capture the associations between miRNAs and diseases. Although the model was based on random walk that made full use of global network information, it was not applicable to new diseases without any known related miRNAs. Chen et al^20^ proposed another global ranking model called WBSMDA, which utilized Gaussian interaction profile kernel similarity for diseases and miRNAs. As an upgrade to RWRMDA, WBSMDA could be implemented for diseases without any known related miRNAs. However, WBSMDA might cause bias to miRNAs with more known associated diseases and its scores needed to be integrated more reasonably. Xuan et al^21^ presented HDMP based on weighted *k* most similar neighbours and the miRNA functional similarity. For a specific disease, the relevance score of a miRNA was calculated by summing all subscores of the miRNA's *k* neighbours. The subscore of a neighbour was calculated by multiplying the functional similarity between the miRNA and the neighbour with the weight of the neighbour; the assignment of weight was based on the neighbour's miRNA family or cluster. The members in the same miRNA family or cluster were assigned higher weights because they were usually transcribed together and therefore were more likely to be associated with similar diseases. However, this method also had some limitations: on one hand, HDMP could not be applied to the new diseases which did not have any known related miRNAs; on the other hand, HDMP did not make full use of global network similarity information. Pasquier et al^22^ proposed a method named MiRAI which represented distributional information on miRNAs and diseases in a high‐dimensional vector space and reduced dimensions with the help of singular value decomposition (SVD). The association score for a miRNA‐disease pair was measured by the cosine similarity between the miRNA vector in the miRNA space and the disease vector in the disease space. However, the prediction accuracy of MiRAI was low because the model had the data sparsity problem.

Besides, several computational models had adopted machine learning methods to uncover associations between miRNAs and diseases. Under the assumption that miRNAs involved in a specific tumour phenotype will exhibit aberrant regulation of their target genes, Xu et al^23^ introduced an approach based on the miRNA‐target‐dysregulated network (MTDN) to prioritize disease‐related miRNAs. MTDN was constructed by assembling all significant miRNA‐target pairs which were identified by miRNA expression profiles in tumour and non‐tumour tissues. For each miRNA in MTDN, 4 topological features were computed and changes in miRNA expression were captured. Then, a support vector machine (SVM) classifier was built to identify positive miRNA‐disease associations. Nevertheless, negative associations needed for training the model were hard to obtain and the prediction of supervised classifier such as SVM could be inaccurate. To address this problem, Chen et al^24^ proposed a semi‐supervised method named RLSMDA. It was developed under the framework of regularized least squares and the basic hypothesis that functionally related miRNAs tend to be related to phenotypically similar diseases. Compared with previous methods, RLSMDA could identify related miRNAs for diseases without any known associated miRNAs. Furthermore, only positive disease‐miRNA association samples were needed to train RLSMDA, and therefore, the model overcame the difficulties in obtaining negative samples faced by several previous studies. But the room for improvement was how to choose the best parameters. Similar to the process of random work, Chen et al^25^ presented another iterative model named HGIMDA to find the optimal solutions based on global network similarity information. A heterogeneous graph was constructed from various disease similarity measures, diverse miRNA similarity measures and the known miRNA‐disease associations. To calculate the association score between a miRNA and a disease, an iterative process was carried out on the heterogeneous graph, summarizing all paths between the miRNA and the disease with the length equal to 3. Xuan et al^26^ developed MIDP to predict potential miRNA candidates for the diseases with known related miRNAs and MIDPE for the diseases without any known related miRNAs. It is worth mentioning that the negative effect of noisy data could be decreased by restarting the walk. Chen et al^27^ further raised RBMMMDA which was the first computational approach for multiple types of miRNA‐disease association prediction. Based on this model, we could obtain not only new miRNA‐disease associations but also their corresponding association types. Recently, Li et al^28^ raised MCMDA based on the observation that the miRNA‐disease association matrix was low‐rank. They filled the candidate samples without known associations with zero and then iteratively updated them with the predictive scores.

As mentioned above, the existing methods have different limitations. For example, miRNA‐target interactions and disease‐genes associations used in some methods are inaccurate or incomplete. Furthermore, many methods could not be applied to disease without any known related miRNAs and many methods were constructed without optimal parameter. Therefore, new effective computational methods are in urgent need. Based on the assumption that functional similar miRNAs tend to be associated with similar diseases and vice versa, we developed the model of Network Distance Analysis for MiRNA‐Disease Association prediction (NDAMDA). MiRNA‐disease associations, miRNA functional similarity network, disease semantic similarity network and Gaussian interaction profile kernel similarity network were integrated in NDAMDA to uncover the potential disease‐miRNA associations. To evaluate the effectiveness of NDAMDA, global and local leave‐one‐out cross‐validation (LOOCV) as well as fivefold cross‐validation was introduced. The AUCs of global and local LOOCV were respectively 0.8920 and 0.8062, and the model obtained the average AUC of 0.8935 ± 0.0009 in fivefold cross‐validation. Besides, we accessed NDAMDA in case studies of breast neoplasms, lymphoma and oesophageal neoplasms with the validation databases of dbDEMC 29 and miR2Disease.^30^ As a result, 43, 36 and 43 of the top 50 candidate miRNAs for these 3 diseases were respectively confirmed by experimental discoveries in recent years. We further evaluated the applicability of our method to the diseases without any known related miRNAs. Prostate neoplasms was taken as the investigated diseases, and its known associated miRNAs for the investigated disease were removed from the training dataset. We found that 43 of the top 50 candidate miRNAs for prostate neoplasms were verified by experimental discoveries. Finally, we obtained 42 confirmed miRNAs in the top 50 candidate miRNAs for hepatocellular carcinoma based on the previous version of HMDD, further suggesting that this model have a good performance on different input dataset.

## 2 MATERIALS AND METHODS

### 2.1 Human miRNA‐disease associations

We downloaded the latest data of human miRNA‐disease from the HMDD database v2.0,^31^ which included 5430 experimentally verified human miRNA‐diseases associations, and it involved 383 diseases and 495 miRNAs. Here, we introduced matrix *Y* ∈ *R*_nm × nd_ to express those associations in a mathematic way and entity $Y\left(i,j\right)$ equalled 1 if miRNA $m\left(i\right)$ was confirmed to be related to disease $d\left(j\right)$, and otherwise 0. In addition, we used nm and nd to denote the number of miRNAs and diseases, respectively.

### 2.2 MiRNA functional similarity

Based on the assumption that functionally similar miRNAs tend to be associated with phenotypically similar diseases, Wang et al^32^ have calculated the miRNA functional similarity score and we downloaded them from http://www.cuilab.cn/files/images/cuilab/misim.zip. We kept the scores in the matrix FS, where the entity $\mathrm{FS}\left(m\left(i\right),m\left(j\right)\right)$ represented the functional similarity between miRNA $m\left(i\right)$ and $m\left(j\right)$.

### 2.3 Disease semantic similarity model 1

We described each disease as a directed acyclic graph (DAG) with the help of the disease MeSH descriptors downloaded from the National Library of Medicine (http://www.nlm.nih.gov).^33^ Taking disease $d\left(i\right)$ as an example, we used $\mathrm{DAG}\left(d\left(i\right),T\left(d\left(i\right)\right),E\left(d\left(i\right)\right)\right)$ to represent it, where $T\left(d\left(i\right)\right)$ was the node set consisted of node D itself and its ancestor nodes, $E\left(d\left(i\right)\right)$ was the corresponding edge set composed of the direct edges from parent nodes to child nodes. Therefore, summing all the contributions from ancestor diseases and disease $d\left(i\right)$ itself, we could calculate the semantic value of disease $d\left(i\right)$ as follows:

(1) $$\mathrm{DV}\left(d\left(i\right)\right)=\sum_{d\in T\left(d\left(i\right)\right)}D_{d\left(i\right)}\left(d\right)$$

(2) $$\left\{\begin{array}{c} D_{d\left(i\right)}\left(d\right)=1,\;if\;d=d\left(i\right)\\ D_{d\left(i\right)}\left(d\right)=\text{max}\left\{\Delta_{*}D_{d\left(i\right)}\left(d^{\prime }\right)|d^{\prime }\;\in children\;of\;d\right\},\;if\;d\neq d\left(i\right) \end{array}\right.$$

where ∆ was the semantic contribution factor. Their own contribution to the semantic value of disease $d\left(i\right)$ was defined as 1; the contribution decreased as the distance between $d\left(i\right)$ and other diseases increased. Therefore, disease terms in the same layer had the same contribution to the semantic value of disease $d\left(i\right)$. We reasoned that 2 diseases sharing larger part of their DAGs were considered to have greater semantic similarity. Here, we defined semantic similarity between $d\left(i\right)$ and $d\left(j\right)$ as follows:

(3) $$\mathrm{SS}1\left(d\left(i\right),d\left(j\right)\right)=\frac{\sum_{t\in T\left(d\left(i\right)\right)\cap T\left(d\left(j\right)\right)}\left(D_{d\left(i\right)}\left(t\right)+D_{d\left(j\right)}\left(t\right)\right)}{\text{DV}\left(d\left(i\right)\right)+\text{DV}\left(d\left(j\right)\right)}$$

### 2.4 Disease semantic similarity model 2

The disease semantic similarity model was unsatisfying in considering that 2 diseases which located in the same layer of DAG($d\left(i\right)$) might appear in different number of disease DAGs. It is obvious that the one appeared more commonly was less specific. Therefore, we developed disease semantic similarity model 2 to complement the old one. We defined the contribution of disease *t* in DAG($d\left(i\right)$) to the semantic value of disease α as follows:

(4) $$D_{d\left(i\right)}^{*}\left(t\right)=-\text{log}\left[\frac{\text{the}\;\text{amount}\;\text{of}\;\text{DAGs}\;\text{including}\;t}{\text{the}\;\text{amount}\;\text{of}\;\text{diseases}}\right]$$

Based on the assumption that 2 diseases sharing larger part of their DAGs are considered to have stronger semantic similarity, we summed all the contributions from ancestor diseases and itself to determine the semantic value DV of disease $d\left(i\right)$ in the similar way as model 1.

(5) $$\text{DV}\left(d\left(i\right)\right)=\sum_{t\in D\left(d\left(i\right)\right)}D_{d\left(i\right)}^{*}\left(t\right)$$

where $D\left(d\left(i\right)\right)$ was the node set in DAG $\left(d\left(i\right)\right)$. The disease semantic similarity matrix SS2 was given by

(6) $$\;\text{SS2}\left(d\left(i\right),d\left(j\right)\right)=\frac{\sum_{t\in T\left(d\left(i\right)\right)\cap T\left(d\left(j\right)\right)}\left(D_{d\left(i\right)}^{*}\left(t\right)+D_{d\left(j\right)}^{*}\left(t\right)\right)}{\text{DV}\left(d\left(i\right)\right)+\text{DV}\left(d\left(j\right)\right)}$$

where $\text{DV}\left(d\left(i\right)\right)$ and $\text{DV}\left(d\left(j\right)\right)$ were the semantic value of $d\left(i\right)$ and $d\left(j\right)$, respectively.

### 2.5 Gaussian interaction profile kernel similarity for diseases

Using the topologic information of known miRNA‐disease association network, we proposed Gaussian interaction profile kernel similarity for diseases based on the assumption that functional similar miRNAs tend to be associated with similar diseases. Here, we used the vector IP to represent the interaction profiles of diseases, and IP was calculated based on the associated information between the disease and each miRNA, that is, the *ith* row of the adjacency matrix *Y*. Then, Gaussian kernel similarity between disease $d\left(i\right)$ and $d\left(j\right)$ was defined based on their interaction profiles as follows:

(7) $$KS_{d}\left(d\left(i\right),d\left(j\right)\right)=\text{exp}\left(-\gamma_{d}\left|\right|\text{IP}\left(d\left(i\right)\right)-\text{IP}\left(d\left(j\right)\right){\left|\right|}^{2}\right)$$

where parameter$\;\gamma_{d}$ was used to control the kernel bandwidth and calculated as follows.

(8) $$\gamma_{d}=\gamma_{d}^{\prime }/\left(1/\text{nd}\sum_{k=1}^{\text{nd}}||\text{IP}\left(d\left(k\right)\right)|{|}^{2}\right)$$

where $\gamma_{d}^{\prime }$ was the original adjustment coefficient and $KS_{d}\left(d\left(i\right),d\left(j\right)\right)$ was the Gaussian interaction profile kernel similarity between disease $d\left(i\right)$ and $d\left(j\right)$.

### 2.6 Gaussian interaction profile kernel similarity for miRNAs

Gaussian interaction profile kernel similarity matrix of miRNA could be calculated in a similar way:

(9) $$KS_{m}\left(m\left(i\right),m\left(j\right)\right)=\text{exp}\left(-\gamma_{m}\left|\right|\text{IP}\left(m\left(i\right)\right)-\text{IP}\left(m\left(j\right)\right){\left|\right|}^{2}\right)$$

(10) $$\gamma_{m}=\gamma_{m}^{\prime }/\left(\frac{1}{\text{nm}}\sum_{k=1}^{\text{nm}}||\text{IP}\left(m\left(k\right)\right)|{|}^{2}\right)$$

$KS_{m}\left(m\left(i\right),m\left(j\right)\right)$ was the Gaussian interaction profile kernel similarity between miRNA $m\left(i\right)$ and $m\left(j\right)$.

### 2.7 Integrated similarity for miRNAs and diseases

Here, integrated miRNA similarity matrix *S*_*m*_ and integrated disease similarity matrix *S*_*d*_ were constructed based on miRNA functional similarity, disease semantic similarity and Gaussian interaction profile kernel similarity. For miRNA pairs and disease pairs that did not have similarity, we used KS_*m*_ and KS_*d*_ to respectively represent the similarity between them. In addition, we used FS to represent the similarity for miRNA pairs that had functional similarity; we used the average of SS1 and SS2 to represent the similarity for disease pairs that had semantic similarity.

(11) $$S_{m}\left(m\left(i\right),m\left(j\right)\right)=\left\{\begin{array}{c} \text{FS}\left(m\left(i\right),m\left(j\right)\right),m\left(i\right)\;and\;m\left(j\right)\;\text{have}\;\text{functional}\;\text{similarity}\\ KS_{m}\left(m\left(i\right),m\left(j\right)\right),\text{otherwise} \end{array}\right.$$

(12) $$S_{d}\left(d\left(i\right),d\left(j\right)\right)=\left\{\begin{array}{c} \frac{\begin{array}{c} \text{SS1}\left(d\left(i\right),d\left(j\right)\right)+\\ \text{SS2}\left(d\left(i\right),d\left(j\right)\right) \end{array}}{2},d\left(i\right)\;\text{and}\;d\left(j\right)\;\text{have}\;\text{semantic}\;\text{similarity}\\ KS_{d}\left(d\left(i\right),d\left(j\right)\right)\;,\text{otherwise} \end{array}\right.$$

### 2.8 NDAMDA

**Figure 1:**
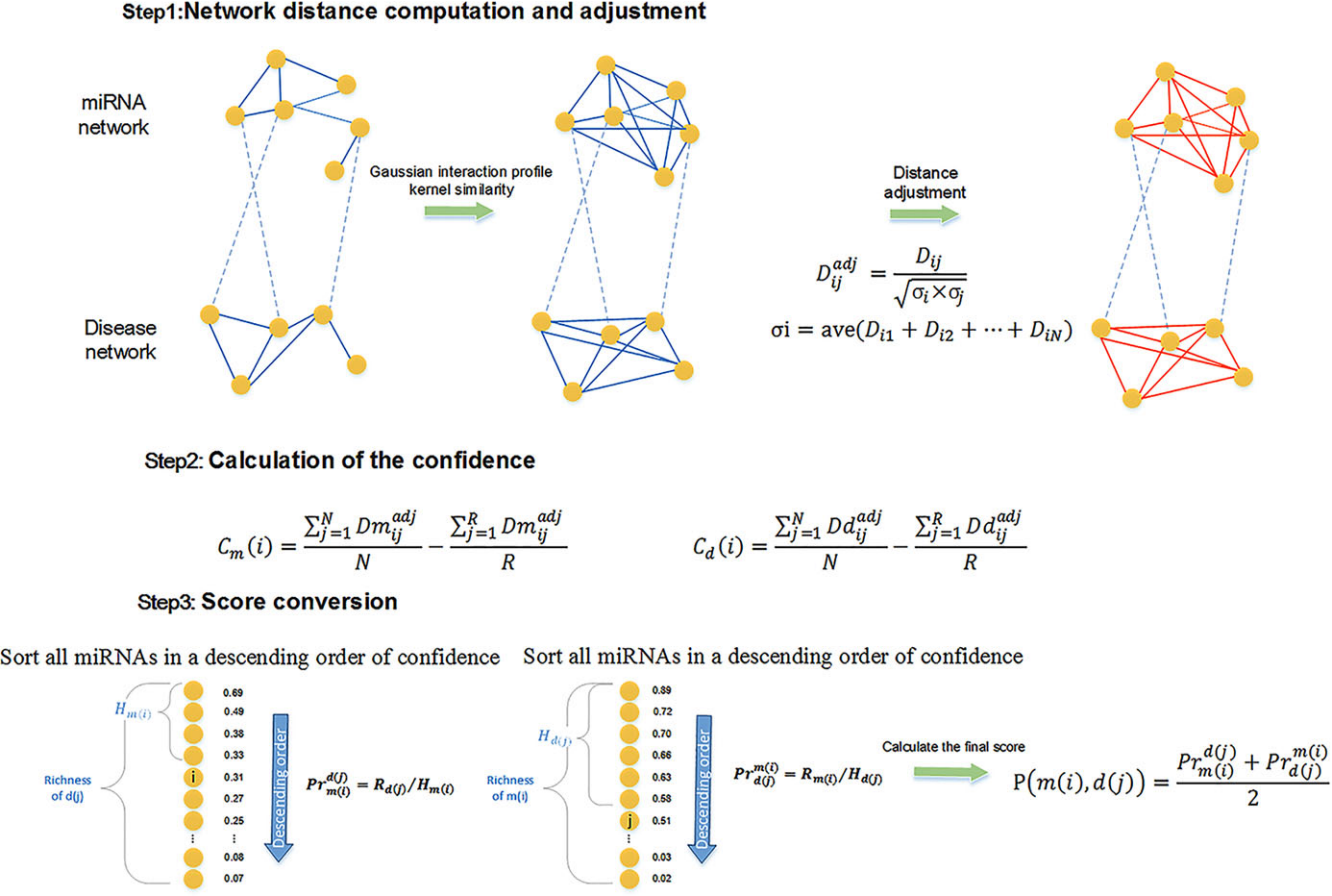
The flow chart of NDAMDA includes 3 steps: (I) network distance computation and adjustment (II) calculation of the confidence (III) score conversion. For details about each step, refer to the Materials and Method section

We developed NDAMDA which was constituted by 3 steps: (I) network distance computation and adjustment (II) calculation of the confidence (III) score conversion (See Figure 1).

### 2.9 Network distance computation and adjustment for miRNAs

We could obtain the similarities from our previous work between two miRNAs directly, for example, we could extract functional similarity between $m\left(i\right)$ and $m\left(j\right)$ as $\text{FS}\left(i,j\right)$, then, the raw network distance between two miRNAs with a link in the network was defined as *D *=* *1/FS, such that a smaller *D* (shorter distance) would correspond to a higher functional similarity. To those miRNAs without direct links, we used Gaussian interaction profile kernel similarity to fill it. In summary, the raw distance was determined as *D *=* *1/*S*_*m*_. To develop a comprehensive network, we considered both the distance between two miRNAs and their respective mean network distances to all other miRNAs, and the adjusted distance was defined as $Dm_{\mathrm{ij}}^{\text{adj}}=\frac{D_{\mathrm{ij}}}{\sqrt{\sigma_{i}\times \sigma_{j}}}$ where σ*i* and σ*j* were the mean distance for $m\left(i\right)$ and $m\left(j\right),$ respectively, in raw network.

### 2.10 Network distance computation and adjustment for diseases

Similar to computation and adjustment for miRNAs, the scores obtained from disease semantic similarity model 1 and disease semantic similarity model 2 were used to construct the raw network of diseases, and distance between two diseases was defined as *D *=* *1/*S*_*d*_ after incorporating Gaussian interaction profile kernel similarity to enhance our network. We calculated the adjusted distance as:$\;Dd_{\mathrm{ij}}^{\mathrm{adj}}=\frac{D_{\mathrm{ij}}}{\sqrt{\sigma_{i}\times \sigma_{j}}}$, where σ*i* and σ*j* were the mean distance for$\;d\left(i\right)$ and $d\left(j\right),$ respectively, in raw network.

### 2.11 Calculation of the confidence in miRNAs

We reasoned that, for a specific disease in the network, a related miRNA was closer to other related miRNAs than random miRNAs. Therefore, we introduced the confidence $C_{m}\left(i\right)$ in miRNA $m\left(i\right)$ as follows:

(13) $$C_{m}\left(i\right)=\frac{\sum_{j=1}^{\text{nm}}Dm_{\mathrm{ij}}^{\text{adj}}}{\text{nm}}-\frac{\sum_{j=1}^{R}Dm_{\mathrm{ij}}^{\text{adj}}}{R}$$

where *R* was richness of the given disease indicating the total number of known related miRNAs, and $Dm_{\mathrm{ij}}^{\mathrm{adj}}$ was the adjusted network distance between $m\left(i\right)$ and $m\left(j\right)$. It could be concluded that a larger $C_{m}\left(i\right)$ would suggest that the investigated miRNA had relatively shorter distance (stronger functional interaction) to known related miRNAs than to random miRNAs; the miRNA was therefore more likely to be associated with the investigated disease.

### 2.12 Calculation of the confidence to pick the diseases regulated by specific miRNA

Similarly, given a specific miRNA, we reasoned that a regulated disease deserves a stronger integrated similarity than with random diseases. Here, we introduced the confidence in $d\left(i\right)$, $C_{d}\left(i\right)$, defined as follows:

(14) $$C_{d}\left(i\right)=\frac{\sum_{j=1}^{\text{nd}}Dd_{\mathrm{ij}}^{\text{adj}}}{\text{nd}}-\frac{\sum_{j=1}^{R}Dd_{\mathrm{ij}}^{\text{adj}}}{R}$$

A larger $C_{d}\left(i\right)$ could suggest that the disease under investigation was more likely to be associated with the given miRNA.

### 2.13 Score conversion

For a given disease, the confidence in specific miRNA could be compared with each other, with higher confidence indicating higher probability to be an associated miRNA. However, they could not be directly compared across diseases, because the richness varied greatly from disease to disease. For example, disease A had 20 known related miRNAs and the investigated miRNA was ranked 205th and disease B had 200 known related miRNAs and the second miRNA we investigated was ranked 205th as well. It was obvious that the second pair was more likely to associate with each other and was more likely to be an associated miRNA. Similarly, it was unreasonable to compare confidence across miRNAs. Therefore, a score conversion procedure would be needed to convert the confidence into probabilities. For a given disease, we firstly sorted all miRNAs in a descending order of confidence calculated by NDAMDA. Then, at each *C*_*m*_(*i*), we computed the corresponding precision defined as Precision = *R*/*H*, where *R* and *H* were the richness of given disease (total number of known related miRNAs) and the number of all miRNAs with higher or equal rank to *C*_*m*_(*i*), respectively. In a similar manner, score conversion for a given miRNA was applied on $C_{d}\left(i\right)$ after sorting all diseases in a descending order.

Finally, we integrated two converted scores by averaging them, yielding the final score:

$$P\left(m\left(i\right),d\left(j\right)\right)=\frac{{\text{Pr}}_{m\left(i\right)}^{d\left(j\right)}+{\text{Pr}}_{d\left(j\right)}^{m\left(i\right)}}{2}$$

where $P\left(m\left(i\right),d\left(j\right)\right)$ was the final score between miRNA $m\left(i\right)$ and disease $d\left(j\right)$. ${\mathrm{Pr}}_{m\left(i\right)}^{d\left(j\right)}$ was the precision of $C_{m}\left(i\right)$ when given disease $d\left(j\right),$ and ${\mathrm{Pr}}_{d\left(j\right)}^{m\left(i\right)}$ was the precision of $C_{d}\left(j\right)$ when given miRNA $m\left(i\right)$.

## 3 RESULT

### 3.1 Performance evaluation

We implemented local and global LOOCV to evaluate the prediction accuracy of NDAMDA and 6 previous computational models: WBSMDA,^20^ RLSMDA,^24^ MCMDA,^28^ HDMP,^21^ RWRMDA 19 and MiRAI.^22^ In LOOCV, each known association was used as the validation sample and the remaining known associations were regarded as the training samples. The miRNA‐disease pairs without any known association evidence were considered as candidate samples. The known miRNA‐disease associations were obtained from the HMDD v2.0 database.^31^ The association scores of all miRNA‐disease pairs would be returned by NDAMDA. In global LOOCV, the score of the validation sample was compared with all the candidate samples, while in local LOOCV, the score was compared with candidate samples for the investigated disease.

In fivefold cross‐validation, the known miRNA‐disease associations were randomly partitioned into 5 equally sized subsets. Each subset was retained as the validation set in turn, and the remaining 4 subsets were used as the training set. Still, the miRNA‐disease pairs without known association evidence were regarded as the candidate samples. Then, the score of each sample in the validation set was ranked against the scores of all the candidate samples. This procedure was repeated 100 times to better estimate the mean and variance of NDAMDA's prediction accuracy. This repetition reduced the error in performance estimation as the result of fivefold cross‐validation depended on how the associations were partitioned.

In both LOOCV and fivefold cross‐validation, the model would be deemed to make a correct prediction for a validation sample, if its rank exceeded a given threshold. Furthermore, we drew receiver operating characteristics (ROC) curve by plotting the true‐positive rate (TPR) against the false‐positive rate (FPR) at various thresholds. The true‐positive rate is also known as sensitivity which represents the percentage of the validation samples ranked higher than the threshold. The false‐positive is calculated as (1‐specificity), where specificity denotes the percentage of candidate miRNA‐disease pairs ranked lower than the threshold. We calculated the area under the ROC curve (AUC) to evaluate the prediction ability of NDAMDA. AUC = 1 indicates the model has perfect prediction performance; AUC = 0.5 implies the model has random performance.

**Figure 2:**
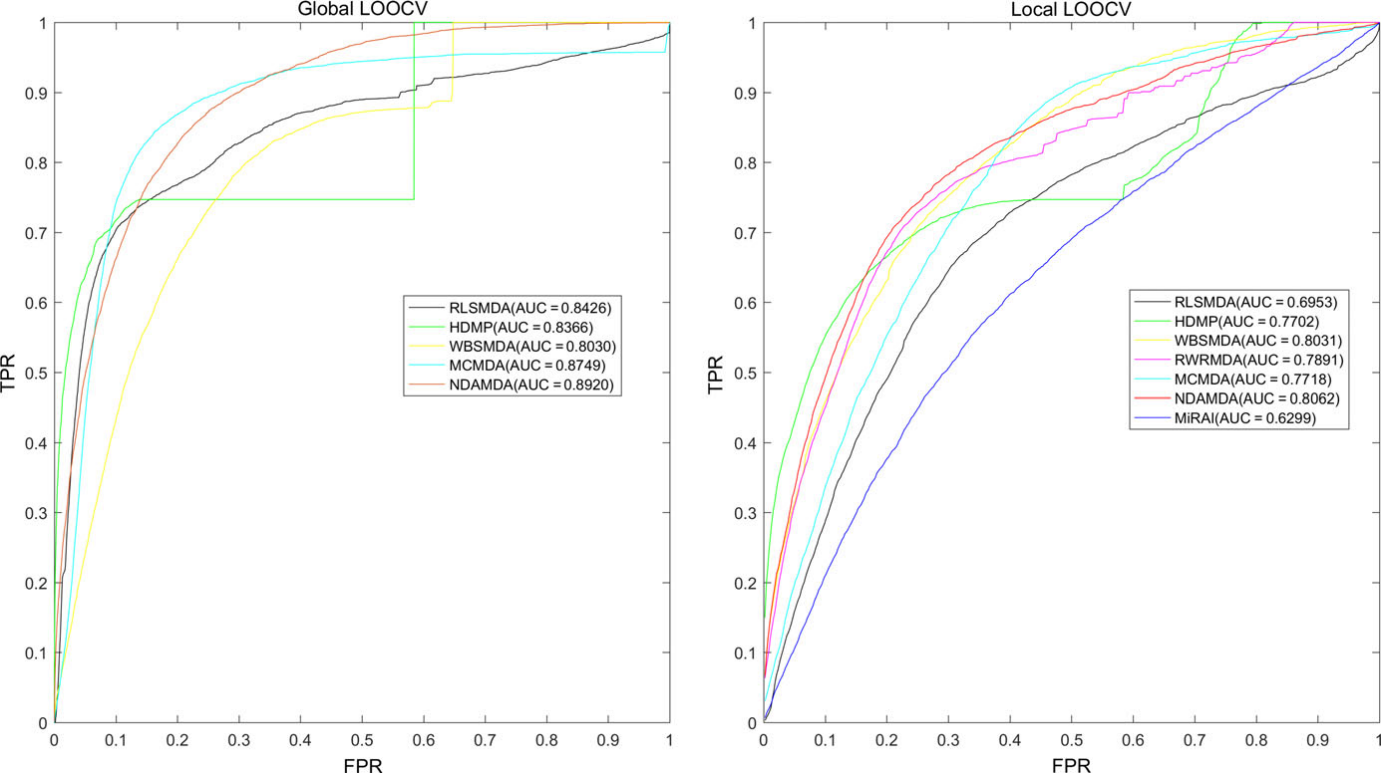
AUC of global LOOCV (left) compared with HGIMDA, RLSMDA, HDMP and WBSMDA; AUC of local LOOCV (right) compared with MCMDA, RLSMDA, HDMP, WBSMDA and MiRAI. As a result, NDAMDA achieved AUCs of 0.8920 and 0.8062 in the global and local LOOCV, which exceed all the previous classical models. LOOCV, leave‐one‐out cross‐validation

The performance comparison in local and global LOOCV is shown in Figure 2. RWRMDA was not included in global LOOCV, because it was a local method, unable to uncover potentially associated miRNAs for all diseases simultaneously. In addition, global LOOCV was not applicable to for MiRAI, because the association score for a miRNA‐disease pair calculated by the model was highly positively correlated with the number of known associated miRNAs for the disease. This means that the association scores for different diseases were not comparable. The cross‐validation results showed that in global LOOCV, the AUCs of NDAMDA, MCMDA, RLSMDA, HDMP, WBSMDA were 0.8920, 0.8749, 0.8426, 0.8366 and 0.8030, respectively; in local LOOCV, NDAMDA, MCMDA, RLSMDA, HDMP, WBSMDA, RWRMDA and MiRAI achieved AUCs of 0.8062, 0.7718, 0.6953, 0.7702, 0.8031, 0.7891 and 0.6299, respectively. In addition, the average AUC of NDAMDA (0.8935 ± 0.0009) exceeded the average AUCs of MCMDA (0.8767 ± 0.0011), RLSMDA (0.8569 ± 0.0020), HDMP (0.8342 ± 0.0010) and WBSMDA (0.8185 ± 0.0009), indicating the superior performance of NDAMDA.

### 3.2 Case studies

To demonstrate the sound prediction accuracy of our method, we further carried out 3 types of case studies on 5 important diseases. In the first type of case studies, the top 10 and top 50 predicted miRNAs for the investigated diseases were validated by another two miRNA‐disease databases, namely dbDEMC 29 and miR2Disease.^30^

Breast cancer is the most commonly diagnosed in females. With more than 1 million new incidences every year, breast cancer is ranked as the second most frequent cancer type when considering both sexes together.^34^ Incidence rates are high in most of the developed areas, and more than half of the cases are in industrialized countries.^35^ It is the leading cause of death in females aged 20‐59.^36^ With the rapid development of high‐throughput sequencing technologies, researchers have identified plenty of miRNAs associated with breast cancer. For example, higher levels of circulating miR‐122 specifically predicted metastatic recurrence in patients with stage II‐III breast cancer.^37^ Besides, miR‐155 was up‐regulated greater than twofold in breast cancer compared with normal adjacent tissue (NAT),^38^ while a decreased level of serum miR‐155 was found after surgery and 4 cycles of chemotherapy.^39^ Here, we implemented NDAMDA to identify potentially related miRNAs for brest neoplasms. As a result, 9 of the top 10 and 42 of the top 50 predicted miRNAs were verified by experimental literatures from dbDEMC and miR2Disease (See Table 1).

**Table 1:** The top 50 predicted miRNAs associated with breast neoplasms by sorting the association probabilities calculated by NDAMDA

miRNA	Evidence	miRNA	Evidence
hsa‐mir‐16	dbdemc	hsa‐mir‐181	Unconfirmed
hsa‐mir‐1247	Unconfirmed	hsa‐mir‐181c	Dbdemc
hsa‐mir‐345	dbdemc	hsa‐mir‐100	Dbdemc
hsa‐mir‐143	dbdemc;miR2Disease	hsa‐let‐7c	Dbdemc
hsa‐mir‐215	dbdemc	hsa‐mir‐1302	Unconfirmed
hsa‐mir‐150	dbdemc	hsa‐mir‐107	Dbdemc
hsa‐mir‐15a	dbdemc	hsa‐mir‐503	Dbdemc
hsa‐mir‐15b	dbdemc	hsa‐mir‐483	Dbdemc
hsa‐mir‐10b	dbdemc;miR2Disease	hsa‐mir‐33a	Unconfirmed
hsa‐mir‐141	dbdemc;miR2Disease	hsa‐mir‐422a	Dbdemc
hsa‐mir‐21	dbdemc;miR2Disease	hsa‐mir‐200c	dbdemc;miR2Disease
hsa‐mir‐198	dbdemc	hsa‐mir‐20a	miR2Disease
hsa‐mir‐590	dbdemc	hsa‐mir‐133a	Dbdemc
hsa‐mir‐200a	dbdemc;miR2!Disease	hsa‐mir‐498	Dbdemc
hsa‐mir‐29a	dbdemc	hsa‐mir‐145	dbdemc;miR2Disease
hsa‐mir‐26b	dbdemc	hsa‐mir‐200b	dbdemc;miR2Disease
hsa‐mir‐675	Unconfirmed	hsa‐let‐7d	dbdemc;miR2Disease
hsa‐mir‐221	dbdemc;miR2Disease	hsa‐let‐7b	Dbdemc
hsa‐mir‐765	dbdemc	hsa‐mir‐942	Unconfirmed
hsa‐let‐7a	dbdemc;miR2Disease	hsa‐mir‐518a	Dbdemc
hsa‐mir‐29b	dbdemc;miR2Disease	hsa‐mir‐181b	dbdemc;miR2Disease
hsa‐mir‐17	miR2Disease	hsa‐mir‐99b	Dbdemc
hsa‐mir‐195	dbdemc;miR2Disease	hsa‐mir‐125a	dbdemc;miR2Disease
hsa‐mir‐1	dbdemc	hsa‐mir‐27b	Dbdemc
hsa‐mir‐103b	Unconfirmed	hsa‐let‐7 g	Dbdemc

The first column records top 1‐25 related miRNAs. The second column records the top 26‐50 related miRNAs.

Oesophageal cancer is the eighth most common cancer worldwide (accounting for about 500 000 new cases every year) and the sixth most common cause of death by cancers (with 400 000 deaths each year).^34^ Moreover, cancer in the oesophagus is usually 3‐4 times more common among males than females and has a very low 5‐year survival rate: only 16% in the United States and 10% in Europe.^35^ Squamous cell carcinoma and adenocarcinoma, the two main types of oesophageal cancer, are mainly caused by overweight, obesity and chronic gastro‐oesophageal reflux disease (GERD).^40^ Researchers have identified several miRNAs associated with oesophagus cancer. For instance, using real‐time RT‐PCR, a research group studied miRNA‐21 levels in serum samples from patients with oesophageal squamous cell carcinomas (ESCC); they found that the patients’ serum concentration of miRNA‐21 was remarkably higher than that of healthy controls and that a significant reduction in the concentration was observed in patients when applied surgery or chemotherapy.^41^, ^42^ Another research group noticed that the expression of exosomal miR‐21 was up‐regulated in serum from patients with ESCC compared with serum from patients with benign diseases.^43^ Among the top 10 and 50 potential oesophageal cancer‐related miRNAs, respectively, 8 and 43 miRNA‐disease‐predicted associations were supported by database evidence (See Table 2).

**Table 2:** The top 50 predicted miRNAs associated with oesophageal neoplasms by sorting the association probabilities calculated by NDAMDA

miRNA	Evidence	miRNA	Evidence
hsa‐mir‐146a	dbdemc	hsa‐mir‐765	Dbdemc
hsa‐mir‐133b	dbdemc	hsa‐mir‐29b	Dbdemc
hsa‐mir‐1247	Unconfirmed	hsa‐mir‐195	Dbdemc
hsa‐mir‐205	dbdemc;miR2Disease	hsa‐let‐7a	Dbdemc
hsa‐mir‐152	dbdemc	hsa‐mir‐17	Dbdemc
hsa‐mir‐345	dbdemc	hsa‐mir‐1	Dbdemc
hsa‐mir‐143	dbdemc	hsa‐mir‐181	Unconfirmed
hsa‐mir‐215	dbdemc	hsa‐mir‐103b	Unconfirmed
hsa‐mir‐148b	dbdemc	hsa‐mir‐181c	dbdemc
hsa‐mir‐449b	Unconfirmed	hsa‐mir‐100	dbdemc
hsa‐mir‐15a	dbdemc	hsa‐mir‐1302	Unconfirmed
hsa‐mir‐150	dbdemc	hsa‐mir‐483	dbdemc
hsa‐mir‐140	dbdemc	hsa‐let‐7c	dbdemc
hsa‐mir‐10b	dbdemc	hsa‐mir‐20a	dbdemc
hsa‐mir‐1972	Unconfirmed	hsa‐mir‐422a	dbdemc
hsa‐mir‐15b	dbdemc	hsa‐mir‐145	dbdemc
hsa‐mir‐21	dbdemc;miR2 Disease	hsa‐mir‐200b	dbdemc
hsa‐mir‐198	dbdemc	hsa‐mir‐107	dbdemc;miR2Disease
hsa‐mir‐141	dbdemc	hsa‐let‐7b	dbdemc
hsa‐mir‐590	dbdemc	hsa‐mir‐133a	dbdemc
hsa‐mir‐200a	dbdemc	hsa‐mir‐200c	dbdemc
hsa‐mir‐29a	dbdemc	hsa‐let‐7d	dbdemc
hsa‐mir‐26b	dbdemc	hsa‐mir‐498	dbdemc
hsa‐mir‐675	Unconfirmed	hsa‐mir‐503	dbdemc
hsa‐mir‐221	dbdemc	hsa‐mir‐181b	dbdemc

The first column records top 1‐25 related miRNAs. The second column records the top 26‐50 related miRNAs.

Lymphoma cancer begins in cells of the immune system and can be divided into two main categories: Hodgkin lymphoma and non‐Hodgkin lymphoma, which accounts for 90 per cent of all lymphomas.^44^, ^45^ Hodgkin lymphoma can be identified by the presence of a type of cell called the Reed‐Sternberg cell, and non‐Hodgkin consists of a large, multiple group of cancers of immune system cells.^46^, ^47^ Recently, researcher found that the expression of miR‐21 in plasma of patient with lymphoma group significantly correlated with their serum LDH level and the higher expressions of miR‐21, miR‐155 and miR‐210 in plasma of patients with lymphoma were significantly higher.^48^ It was also reported that miR‐203, miR‐218, miR‐181a, miR19a and miR17 were found to be associated with lymphoma: the former 3 miRNAs functioned as tumour suppressors, and the latter two were found to up‐regulate oncogenes for lymphoma.^49^ This finding coincided with the generally accepted idea that canine lymphoma is a common spontaneous tumour with great similarities to human lymphoma.^50^ Similarly, we used dbDEMC and miR2Disease to validate the potentially associated miRNAs for lymphoma, and 9 of the top 10 and 36 of the top 50 candidate miRNAs were examined by two databases (See Table 3).

**Table 3:** The top 50 predicted miRNAs associated with lymphoma by sorting the association probabilities calculated by NDAMDA

miRNA	Evidence	miRNA	Evidence
hsa‐mir‐1247	Unconfirmed	hsa‐mir‐133a	dbdemc
hsa‐mir‐215	dbdemc	hsa‐mir‐151	miR2Disease
hsa‐mir‐15b	dbdemc	hsa‐mir‐376c	Unconfirmed
hsa‐mir‐10b	dbdemc	hsa‐mir‐181d	dbdemc
hsa‐mir‐21	dbdemc;miR2Disease	hsa‐mir‐23a	dbdemc
hsa‐mir‐200a	dbdemc	hsa‐mir‐659	Unconfirmed
hsa‐let‐7a	dbdemc	hsa‐let‐7d	dbdemc
hsa‐mir‐26b	dbdemc	hsa‐mir‐422a	dbdemc
hsa‐mir‐221	dbdemc;miR2Disease	hsa‐mir‐10a	dbdemc;miR2Disease
hsa‐mir‐17	dbdemc;miR2Disease	hsa‐mir‐483	Unconfirmed
hsa‐mir‐33a	dbdemc	hsa‐mir‐149	dbdemc;miR2Disease
hsa‐mir‐195	dbdemc	hsa‐mir‐193b	Unconfirmed
hsa‐mir‐503	dbdemc	hsa‐mir‐301b	Unconfirmed
hsa‐mir‐130a	dbdemc	hsa‐mir‐1323	Unconfirmed
hsa‐mir‐103b	Unconfirmed	hsa‐let‐7b	dbdemc
hsa‐mir‐1	dbdemc	hsa‐mir‐20a	dbdemc;miR2Disease
hsa‐mir‐181c	dbdemc	hsa‐mir‐26a	dbdemc
hsa‐mir‐107	dbdemc	hsa‐let‐7 g	dbdemc
hsa‐let‐7c	dbdemc	hsa‐mir‐31	dbdemc
hsa‐mir‐29b	dbdemc	hsa‐mir‐181b	dbdemc
hsa‐mir‐498	Unconfirmed	hsa‐mir‐410	Unconfirmed
hsa‐mir‐200c	dbdemc	hsa‐mir‐125a	dbdemc
hsa‐mir‐1302	Unconfirmed	hsa‐mir‐200b	dbdemc
hsa‐mir‐942	Unconfirmed	hsa‐mir‐204	dbdemc
hsa‐mir‐518a	Unconfirmed	hsa‐mir‐433	Unconfirmed

The first column records top 1‐25 related miRNAs. The second column records the top 26‐50 related miRNAs.

In addition to the above 3 cancers, we used NDAMDA to prioritize candidate miRNAs for all diseases in HMDD v2.0 and the results are included in Table S1.

To assess the ability of NDAMDA in predicting potentially related miRNAs for diseases without any known associated miRNAs, we carried out another case study on prostate cancer. Its associated miRNAs were removed from the training set, and the rest known miRNA‐disease associations were used to train NDAMDA. In this manner, potentially related miRNAs for prostate cancer were uncovered only using the information of other diseases‐related miRNAs and the similarity measures. Subsequently, the top 50 prediction outcomes were confirmed with HMDD v2.0, dbDEMC and miR2Disease. As shown in Table 4, 8 of the top 10, 18 of the top 20 and 43 of the top 50 candidate miRNAs were verified.

**Table 4:** The top 50 predicted miRNAs associated with prostate neoplasms by sorting the association probabilities calculated by NDAMDA

miRNA	Evidence	miRNA	Evidence
hsa‐mir‐21	dbdemc;miR2Disease	hsa‐mir‐31	dbdemc;miR2Disease
hsa‐mir‐155	dbdemc	hsa‐mir‐199a	dbdemc;miR2Disease
hsa‐mir‐146a	miR2Disease	hsa‐mir‐9	dbdemc
hsa‐mir‐125b	dbdemc;miR2Disease;HMDD	hsa‐mir‐181a	dbdemc;miR2Disease
hsa‐mir‐17	miR2Disease	hsa‐mir‐133a	dbdemc
hsa‐mir‐20a	miR2Disease	hsa‐mir‐210	miR2Disease
hsa‐mir‐34a	dbdemc;miR2Disease	hsa‐let‐7b	dbdemc;miR2Disease
hsa‐mir‐145	dbdemc;miR2Disease;HMDD	hsa‐mir‐200a	dbdemc
hsa‐mir‐221	dbdemc;miR2Disease	hsa‐mir‐200c	dbdemc
hsa‐mir‐18a	Unconfirmed	hsa‐mir‐181b	dbdemc;miR2Disease
hsa‐mir‐16	dbdemc;miR2Disease	hsa‐mir‐142	Unconfirmed
hsa‐mir‐92a	Unconfirmed	hsa‐mir‐150	dbdemc
hsa‐mir‐126	dbdemc;miR2Disease	hsa‐mir‐34c	dbdemc
hsa‐mir‐19b	dbdemc;miR2Disease	hsa‐let‐7c	dbdemc;miR2Disease
hsa‐mir‐15a	dbdemc;miR2Disease	hsa‐mir‐146b	Unconfirmed
hsa‐mir‐19a	dbdemc	hsa‐mir‐122	Unconfirmed
hsa‐mir‐29a	dbdemc;miR2Disease	hsa‐mir‐106b	dbdemc
hsa‐mir‐1	dbdemc	hsa‐mir‐182	dbdemc;miR2Disease
hsa‐mir‐222	dbdemc;miR2Disease	hsa‐let‐7d	dbdemc;miR2Disease
hsa‐mir‐143	dbdemc;miR2Disease	hsa‐mir‐141	miR2Disease
hsa‐mir‐29b	dbdemc;miR2Disease	hsa‐let‐7e	dbdemc
hsa‐let‐7a	dbdemc;miR2Disease	hsa‐mir‐133b	dbdemc
hsa‐mir‐200b	Unconfirmed	hsa‐mir‐214	dbdemc;miR2Disease
hsa‐mir‐223	dbdemc;miR2Disease	hsa‐mir‐203	Unconfirmed
hsa‐mir‐29c	dbdemc	hsa‐mir‐30a	miR2Disease

The disease's associated miRNAs were removed from the training set, and the rest known miRNA‐disease associations were used to train NDAMDA. Subsequently, the top 50 prediction outcomes were confirmed with HMDD v2.0, dbDEMC and miR2Disease. The first column records top 1‐25 related miRNAs. The second column records the top 26‐50 related miRNAs.

In the final case study, we fitted our model with the miRNA‐disease association dataset from HMDD v1.0, the old version of the HMDD database. This case study was meant to demonstrate NDAMDA's robust prediction ability to various datasets. Hepatocellular carcinoma was chosen as the investigated disease; its potentially associated miRNAs were identified by NDAMDA and validated against HMDD v2.0, dbDEMC and miR2Disease. As a result, 42 of the top 50 candidates were confirmed by experimental evidence from the databases (See Table 5). Taking the 1st candidate miR‐155 as an example, it inhibited HBV infection in human hepatoma cells through enhancing innate antiviral immunity; the ectopic expression of miR‐155 up‐regulated the expression of several IFN‐inducible antiviral genes in human hepatoma cells.^51^

**Table 5:** The top 50 predicted miRNAs associated with hepatocellular carcinoma by sorting the association probabilities calculated by NDAMDA

miRNA	Evidence	miRNA	Evidence
hsa‐mir‐155	miR2Disease;HMDD	hsa‐mir‐133a	miR2Disease
hsa‐mir‐16	miR2Disease;HMDD	hsa‐mir‐150	miR2Disease;HMDD
hsa‐mir‐208	Unconfirmed	hsa‐mir‐24	miR2Disease;HMDD
hsa‐let‐7a	miR2Disease;HMDD	hsa‐mir‐132	miR2Disease
hsa‐mir‐15a	miR2Disease;HMDD	hsa‐mir‐141	miR2Disease;HMDD
hsa‐mir‐598	Unconfirmed	hsa‐mir‐9	miR2Disease
hsa‐mir‐539	Unconfirmed	hsa‐mir‐29c	HMDD
hsa‐mir‐550	Unconfirmed	hsa‐mir‐15b	HMDD
hsa‐mir‐652	Unconfirmed	hsa‐mir‐181a	miR2Disease;HMDD
hsa‐let‐7b	miR2Disease;HMDD	hsa‐mir‐210	HMDD
hsa‐mir‐328	miR2Disease	hsa‐mir‐30c	miR2Disease;HMDD
hsa‐let‐7c	miR2Disease;HMDD	hsa‐mir‐107	miR2Disease;HMDD
hsa‐let‐7i	HMDD	hsa‐mir‐194	miR2Disease
hsa‐let‐7d	miR2Disease;HMDD	hsa‐mir‐30d	HMDD
hsa‐mir‐411	Unconfirmed	hsa‐mir‐373	HMDD
hsa‐mir‐29b	HMDD	hsa‐mir‐205	miR2Disease;HMDD
hsa‐mir‐143	miR2Disease	hsa‐mir‐30a	miR2Disease;HMDD
hsa‐mir‐181b	miR2Disease;HMDD	hsa‐mir‐200c	HMDD
hsa‐mir‐126	miR2Disease;HMDD	hsa‐mir‐25	miR2Disease;HMDD
hsa‐let‐7 g	miR2Disease;HMDD	hsa‐mir‐196a	HMDD
hsa‐let‐7f	miR2Disease;HMDD	hsa‐mir‐191	HMDD
hsa‐mir‐146b	HMDD	hsa‐mir‐32	Unconfirmed
hsa‐mir‐106b	miR2Disease;HMDD	hsa‐mir‐93	miR2Disease;HMDD
hsa‐mir‐29a	HMDD	hsa‐mir‐451	Unconfirmed
hsa‐mir‐214	miR2Disease;HMDD	hsa‐mir‐34c	HMDD

The model was fitted by the miRNA‐disease association dataset from HMDD v1.0, the old version of the HMDD database. Potentially associated miRNAs for the disease were validated against HMDD v2.0, dbDEMC and miR2Disease. The first column records top 1‐25 related miRNAs. The second column records the top 26‐50 related miRNAs.

## 4 DISCUSSIONS

The experimental methods for identifying disease‐miRNA associations are expensive and time‐consuming. Encouragingly, plenty of computational methods for predicting disease‐related miRNAs have been proposed in recent years. To predict potentially related miRNAs for diseases at a higher accuracy than previous methods, we developed a network analysis‐based method named NDAMDA for prioritizing potentially disease‐related miRNAs. The model achieved sound prediction performance throughout global and local LOOCV, fivefold cross‐validation and 3 types of case studies on 5 major human diseases. Therefore, NDAMDA would be a useful resource for researches to discover associations between diseases and miRNAs.

In our work, the case studies were based on cancers. The hallmarks of cancer are one of the most widely acknowledged organizing principles for research on cancer, and currently, ten hallmarks have been identified to represent the acquired capabilities that distinguish cancer from normal tissue.^52^ These hallmarks are (1) self‐sufficiency in growth signals; (2) insensitivity to antigrowth signals; (3) evading apoptosis; (4) limitless replicative potential; (5) sustained angiogenesis; (6) tissue invasion and metastasis; (7) abnormal metabolic pathways; (8) evading the immune system; (9) chromosome abnormalities and unstable DNA; and (10) inflammation.^52^ Association between cancer hallmarks and genes has been indicated by the literatures.^52^, ^53^, ^54^ For example, in our work, miR‐155 obtained the highest score in the fifth case study on hepatocellular carcinoma; according to the data from NanoString's Hallmarks of Cancer Panel collection (https://www.nanostring.com/), two of the miRNA's gene targets, MUS81 and FLT1, have been found to be associated with Hallmark (9) and Hallmark (5), respectively. Other examples include miR‐16, miR‐1247 and miR‐21, which had the highest scores in the first, third and fourth case studies, respectively. APP, ATG12 and ATF2 are the common targets for these 3 miRNAs and have been identified to be associated with Hallmark (10). In future work, we would consider to involve the information of cancer hallmark‐gene associations in our analysis and examine whether this information could enhance the accuracy of our algorithm.

The reliable performance of NDAMDA could be attributed to several factors as follows. Firstly, heterogeneous datasets (disease‐miRNA associations from HMDD, miRNA functional similarity, disease semantic similarity and Gaussian interaction profile kernel similarity for diseases and miRNAs) were integrated to construct the informative network for prediction. Secondly, we used the adjusted network distance and the algorithm for calculating the confidence in a specific miRNA and the confidence in a specific disease. Finally, we used a score conversion procedure that considered the variation in the number of related miRNAs for different diseases.

Yet, there still exist limitations in NDAMDA. Firstly, more known miRNA‐disease associations are necessary for building a more accurate adjacency network and improving the performance of NDAMDA. Secondly, the model might cause bias to miRNAs with more known related diseases, as it was based on the assumption that the functional similar miRNAs are more likely to be connected with similar diseases. Thirdly, NDAMDA might be not applicable to the diseases whose associated miRNAs tend to distribute randomly in the network, and how to integrate two scores to calculate the final score in a more reasonable way should be studied in future. Finally, although NDAMDA exhibited a commendable predictive performance with the currently available 5430 associations between 495 miRNAs and 383 diseases from HMDD v2.0, this association dataset was still limited; it contained a large amount of unlabelled data and only a very small amount (2.86%) of labelled data, which negatively affected the prediction accuracy. As experimental research continues, more miRNA‐disease associations were expected to be biologically verified in future. With an improved association dataset, our model would be able to uncover disease‐related miRNAs at an even higher accuracy.

## CONFLICT OF INTERESTS

The authors declare no conflict of interests.

## References

[1] Lefebvre L , Sol D . Brains, lifestyles and cognition: are there general trends? Brain Behav Evol. 2008;72:135‐144. 18836259 10.1159/000151473

[2] Bartel DP . MicroRNAs: genomics, biogenesis, mechanism, and function. Cell 2004;116:281‐297. 14744438 10.1016/s0092-8674(04)00045-5

[3] Vonk J , Shackelford TK . The Oxford handbook of comparative evolutionary psychology In: Nathan PE, ed. Oxford Library of Psychology. New York: Oxford University Press; 2012:574.

[4] Pasquinelli AE , Ruvkun G . Control of developmental timing by microRNAs and their targets. Annu Rev Cell Dev Biol. 2002;18:495‐513. 12142272 10.1146/annurev.cellbio.18.012502.105832

[5] Kozomara A , Griffiths‐Jones S . miRBase: annotating high confidence microRNAs using deep sequencing data. Nucleic Acids Res. 2014;42:D68‐D73. 24275495 10.1093/nar/gkt1181 PMC3965103

[6] Ambros V . MicroRNA pathways in flies and worms: growth, death, fat, stress, and timing. Cell. 2003;113:673‐676. 12809598 10.1016/s0092-8674(03)00428-8

[7] Taganov KD , Boldin MP , Chang K‐J , Baltimore D . NF‐κB‐dependent induction of microRNA miR‐146, an inhibitor targeted to signaling proteins of innate immune responses. Proc Natl Acad Sci. 2006;103:12481‐12486. 16885212 10.1073/pnas.0605298103 PMC1567904

[8] Chen J‐F , Mandel EM , Thomson JM , et al. The role of microRNA‐1 and microRNA‐133 in skeletal muscle proliferation and differentiation. Nat Genet. 2006;38:228‐233. 16380711 10.1038/ng1725 PMC2538576

[9] Chen C‐Z , Li L , Lodish HF , Bartel DP . MicroRNAs modulate hematopoietic lineage differentiation. Science. 2004;303:83‐86. 14657504 10.1126/science.1091903

[10] Carleton M , Cleary MA , Linsley PS . MicroRNAs and cell cycle regulation. Cell Cycle. 2007;6:2127‐2132. 17786041 10.4161/cc.6.17.4641

[11] Urbich C , Kuehbacher A , Dimmeler S . Role of microRNAs in vascular diseases, inflammation, and angiogenesis. Cardiovasc Res. 2008;79:581‐588. 18550634 10.1093/cvr/cvn156

[12] Petrocca F , Visone R , Onelli MR , et al. E2F1‐regulated microRNAs impair TGFβ‐dependent cell‐cycle arrest and apoptosis in gastric cancer. Cancer Cell. 2008;13:272‐286. 18328430 10.1016/j.ccr.2008.02.013

[13] Leung AK , Sharp PA . MicroRNA functions in stress responses. Mol Cell. 2010;40:205‐215. 20965416 10.1016/j.molcel.2010.09.027 PMC2996264

[14] Farazi TA , Hoell JI , Morozov P , Tuschl T . MicroRNAs in human cancer. Adv Exp Med Biol. 2013;774:1‐20. 23377965 10.1007/978-94-007-5590-1_1 PMC3704221

[15] Iorio MV , Ferracin M , Liu CG , et al. MicroRNA gene expression deregulation in human breast cancer. Cancer Res. 2005;65:7065‐7070. 16103053 10.1158/0008-5472.CAN-05-1783

[16] Jiang Q , Hao Y , Wang G , et al. Prioritization of disease microRNAs through a human phenome‐microRNAome network. BMC Syst Biol. 2010;4:S2. 10.1186/1752-0509-4-S1-S2 PMC2880408 20522252

[17] Mork S , Pletscher‐Frankild S , Palleja Caro A , Gorodkin J , Jensen LJ . Protein‐driven inference of miRNA‐disease associations. Bioinformatics. 2014;30:392‐397. 24273243 10.1093/bioinformatics/btt677 PMC3904518

[18] Shi H , Xu J , Zhang G , et al. Walking the interactome to identify human miRNA‐disease associations through the functional link between miRNA targets and disease genes. BMC Syst Biol. 2013;7:101. 24103777 10.1186/1752-0509-7-101 PMC4124764

[19] Chen X , Liu M‐X , Yan G‐Y . RWRMDA: predicting novel human microRNA–disease associations. Mol BioSyst. 2012;8:2792‐2798. 22875290 10.1039/c2mb25180a

[20] Chen X , Yan CC , Zhang X , et al. WBSMDA: within and between score for MiRNA‐disease association prediction. Sci Rep. 2016;6:21106. 26880032 10.1038/srep21106 PMC4754743

[21] Xuan P , Han K , Guo M , et al. Prediction of microRNAs associated with human diseases based on weighted k most similar neighbors. PLoS ONE. 2013;8:e70204. 23950912 10.1371/journal.pone.0070204 PMC3738541

[22] Pasquier C , Gardes J . Prediction of miRNA‐disease associations with a vector space model. Sci Rep. 2016;6:27036. 27246786 10.1038/srep27036 PMC4887905

[23] Xu J , Li C‐X , Lv J‐Y , et al. Prioritizing candidate disease miRNAs by topological features in the miRNA target–dysregulated network: case study of prostate cancer. Mol Cancer Ther. 2011;10:1857‐1866. 21768329 10.1158/1535-7163.MCT-11-0055

[24] Chen X , Yan G‐Y . Semi‐supervised learning for potential human microRNA‐disease associations inference. Sci Rep. 2014;4:5501. 24975600 10.1038/srep05501 PMC4074792

[25] Chen X , Yan CC , Zhang X , You Z‐H , Huang Y‐A , Yan G‐Y . HGIMDA: heterogeneous graph inference for miRNA‐disease association prediction. Oncotarget. 2016;7:65257‐65269. 27533456 10.18632/oncotarget.11251 PMC5323153

[26] Xuan P , Han K , Guo Y , et al. Prediction of potential disease‐associated microRNAs based on random walk. Bioinformatics. 2015;31:1805‐1815. 25618864 10.1093/bioinformatics/btv039

[27] Chen X , Yan CC , Zhang X , et al. RBMMMDA: predicting multiple types of disease‐microRNA associations. Sci Rep. 2015;5:13877. 26347258 10.1038/srep13877 PMC4561957

[28] Li JQ , Rong ZH , Chen X , Yan GY , You ZH . MCMDA: matrix completion for MiRNA‐disease association prediction. Oncotarget. 2017;8:21187‐21199. 28177900 10.18632/oncotarget.15061 PMC5400576

[29] Yang Z , Ren F , Liu C , et al. dbDEMC: a database of differentially expressed miRNAs in human cancers. BMC Genom. 2010;11:S5. 10.1186/1471-2164-11-S4-S5 PMC3005935 21143814

[30] Jiang Q , Wang Y , Hao Y , et al. miR2Disease: a manually curated database for microRNA deregulation in human disease. Nucleic Acids Res. 2009;37:D98‐D104. 18927107 10.1093/nar/gkn714 PMC2686559

[31] Li Y , Qiu C , Tu J , et al. HMDD, v2.0: a database for experimentally supported human microRNA and disease associations. Nucleic Acids Res. 2014;42:D1070‐D1074. 24194601 10.1093/nar/gkt1023 PMC3964961

[32] Wang D , Wang J , Lu M , Song F , Cui Q . Inferring the human microRNA functional similarity and functional network based on microRNA‐associated diseases. Bioinformatics. 2010;26:1644‐1650. 20439255 10.1093/bioinformatics/btq241

[33] Lipscomb CE . Medical subject headings (MeSH). Bull Med Libr Assoc. 2000;88:265. 10928714 PMC35238

[34] Jemal A , Ward EM , Johnson CJ , et al. Annual report to the nation on the status of cancer, 1975‐2014, featuring survival. J Natl Cancer Inst 2017;109: https://doi.org/10.1093/jnci/djx030. 10.1093/jnci/djx030 PMC5409140 28376154

[35] Parkin DM , Bray F , Ferlay J , Pisani P . Global cancer statistics, 2002. CA Cancer J Clin. 2005;55:74‐108. 15761078 10.3322/canjclin.55.2.74

[36] Siegel RL , Miller KD , Jemal A . Cancer statistics, 2015. CA Cancer J Clin. 2015;65:5‐29. 25559415 10.3322/caac.21254

[37] Wu X , Somlo G , Yu Y , et al. De novo sequencing of circulating miRNAs identifies novel markers predicting clinical outcome of locally advanced breast cancer. J Transl Med. 2012;10:42. 22400902 10.1186/1479-5876-10-42 PMC3342150

[38] Liu J , Mao Q , Liu Y , Hao X , Zhang S , Zhang J . Analysis of miR‐205 and miR‐155 expression in the blood of breast cancer patients. Chin J Cancer Res 2013;25:46‐54. 23372341 10.3978/j.issn.1000-9604.2012.11.04 PMC3555294

[39] Sun Y , Wang M , Lin G , et al. Serum microRNA‐155 as a potential biomarker to track disease in breast cancer. PLoS ONE. 2012;7:e47003. 23071695 10.1371/journal.pone.0047003 PMC3468565

[40] Torre LA , Bray F , Siegel RL , Ferlay J , Lortet‐Tieulent J , Jemal A . Global cancer statistics, 2012. CA Cancer J Clin. 2015;65:87‐108. 25651787 10.3322/caac.21262

[41] Cai EH , Gao YX , Wei ZZ , Chen WY , Yu P , Li K . Serum miR‐21 expression in human esophageal squamous cell carcinomas. Asian Pac J Cancer Prev. 2012;13:1563‐1567. 22799367 10.7314/apjcp.2012.13.4.1563

[42] Kurashige J , Kamohara H , Watanabe M , et al. Serum microRNA‐21 is a novel biomarker in patients with esophageal squamous cell carcinoma. J Surg Oncol. 2012;106:188‐192. 22354855 10.1002/jso.23064

[43] Tanaka Y , Kamohara H , Kinoshita K , et al. Clinical impact of serum exosomal microRNA‐21 as a clinical biomarker in human esophageal squamous cell carcinoma. Cancer. 2013;119:1159‐1167. 23224754 10.1002/cncr.27895

[44] Gall EA , Mallory TB . Malignant lymphoma: a clinico‐pathologic survey of 618 cases. Am J Pathol. 1942;18:381‐429. 19970634 PMC2032954

[45] Le Count ER . Lymphoma, a benign tumor representing a lymph gland in structure. J Exp Med. 1899;4:559‐567. 10.1084/jem.4.5-6.559 PMC2118014 19866924

[46] Armitage JO , Gascoyne RD , Lunning MA , Cavalli F . Non‐hodgkin lymphoma. Lancet. 2017;390:298‐310. 28153383 10.1016/S0140-6736(16)32407-2

[47] Stein H , Mason DY , Gerdes J , et al. The expression of the Hodgkin's disease associated antigen Ki‐1 in reactive and neoplastic lymphoid tissue: evidence that Reed‐Sternberg cells and histiocytic malignancies are derived from activated lymphoid cells. Blood. 1985;66:848‐858. 3876124

[48] Ge TT , Liang Y , Fu R , et al. [Expressions of miR‐21, miR‐155 and miR‐210 in plasma of patients with lymphoma and its clinical significance]. Zhongguo shi yan xue ye xue za zhi. 2012;20:305‐309. 22541087

[49] Uhl E , Krimer P , Schliekelman P , Tompkins SM , Suter S . Identification of altered MicroRNA expression in canine lymphoid cell lines and cases of B‐ and T‐Cell lymphomas. Genes Chromosom Cancer. 2011;50:950‐967. 21910161 10.1002/gcc.20917

[50] Khanna C , Lindblad‐Toh K , Vail D , et al. The dog as a cancer model. Nat Biotechnol. 2006;24:1065‐1066. 10.1038/nbt0906-1065b 16964204

[51] Su C , Hou Z , Zhang C , Tian Z , Zhang J . Ectopic expression of microRNA‐155 enhances innate antiviral immunity against HBV infection in human hepatoma cells. Virol J. 2011;8:354. 21762537 10.1186/1743-422X-8-354 PMC3169510

[52] Wang E , Zaman N , McGee S , Milanese JS , Masoudi‐Nejad A , O'Connor‐McCourt M . Predictive genomics: a cancer hallmark network framework for predicting tumor clinical phenotypes using genome sequencing data. Semin Cancer Biol. 2015;30:4‐12. 24747696 10.1016/j.semcancer.2014.04.002

[53] Gao S , Tibiche C , Zou J , et al. Identification and construction of combinatory cancer hallmark‐based gene signature sets to predict recurrence and chemotherapy benefit in stage II colorectal cancer. JAMA Oncol. 2016;2:37‐45. 26502222 10.1001/jamaoncol.2015.3413

[54] Fu C , Li J , Wang E . Signaling network analysis of ubiquitin‐mediated proteins suggests correlations between the 26S proteasome and tumor progression. Mol BioSyst. 2009;5:1809‐1816. 19593471 10.1039/B905382D

