# Supplementary material for: NDAMDA: Network distance analysis for MiRNA‐disease association prediction
Source: J Cell Mol Med. 2018 Mar 13;22(5):2884–95. doi: 10.1111/jcmm.13583 (PMC5908143; doi:10.1111/jcmm.13583)
Supplement: Supplementary file 2 [file JCMM-22-2884-s002.docx]

NDAMDA: Network Distance Analysis for MiRNA-Disease Association prediction

Xing Chen^1,#,*^, Le-Yi Wang^2,#^ , Li Huang^3^

^1^School of Information and Control Engineering, China University of Mining and Technology, Xuzhou, 221116, China

^2^School of Mathematics and Statistics, Wuhan University, Luojiashan, Wuchang, 430072, China

^3^ Business Analytics Centre, National University of Singapore, 119613, Singapore

*Corresponding author

#The authors wish it to be known that, in their opinion, the first two authors should be regarded as joint First Authors.

**Email**: [xingchen@amss.ac.cn](mailto:xingchen@amss.ac.cn)

**Keywords**: microRNA; disease; association prediction; adjusted network distance; network integration

**Supplementary Information**

**Supplementary Table 1.** We applied NDAMDA to prioritize all the candidate miRNA-disease pairs based on all the known miRNA-disease associations in HMDD database as training samples. This prediction result is released for further experimental validation and research.
